# Multi-Family Pediatric Pain Group Therapy: Capturing Acceptance and Cultivating Change

**DOI:** 10.3390/children4120106

**Published:** 2017-12-07

**Authors:** Samantha E. Huestis, Grace Kao, Ashley Dunn, Austin T. Hilliard, Isabel A. Yoon, Brenda Golianu, Rashmi P. Bhandari

**Affiliations:** 1 Department of Anesthesiology, Perioperative, and Pain Medicine, Stanford University School of Medicine, Stanford, CA 94305, USA; iayoon@stanford.edu (I.A.Y.); bgolianu@stanford.edu (B.G.); rbhandar@stanford.edu (R.P.B.); 2 Departments of Pediatrics and Anesthesiology, Baylor College of Medicine and Texas Children’s Hospital, Houston, TX 77030, USA; grace.kao@bcm.edu; 3 Spectrum, The Stanford Center for Clinical and Translational Research and Education, Palo Alto, CA 94304, USA; adunn2@stanford.edu; 4 Department of Biology, Stanford University; Stanford, CA 94305, USA; ahilliar@stanford.edu

**Keywords:** pediatric chronic pain, children, adolescents, group therapy, cognitive-behavioral therapy (CBT), acceptance and commitment therapy (ACT), multi-family therapy (MFT)

## Abstract

Behavioral health interventions for pediatric chronic pain include cognitive-behavioral (CBT), acceptance and commitment (ACT), and family-based therapies, though literature regarding multi-family therapy (MFT) is sparse. This investigation examined the utility and outcomes of the Courage to Act with Pain: Teens Identifying Values, Acceptance, and Treatment Effects (CAPTIVATE) program, which included all three modalities (CBT, ACT, MFT) for youth with chronic pain and their parents. Program utility, engagement, and satisfaction were evaluated via quantitative and qualitative feedback. Pain-specific psychological, behavioral, and interpersonal processes were examined along with outcomes related to disability, quality of life, pain interference, fatigue, anxiety, and depressive symptoms. Participants indicated that CAPTIVATE was constructive, engaging, and helpful for social and family systems. Clinical and statistical improvements with large effect sizes were captured for pain catastrophizing, acceptance, and protective parenting but not family functioning. Similar effects were found for functional disability, pain interference, fatigue, anxiety, and depression. Given the importance of targeting multiple systems in the management of pediatric chronic pain, preliminary findings suggest a potential new group-based treatment option for youth and families. Next steps involve evaluating the differential effect of the program over treatment as usual, as well as specific CBT, ACT, and MFT components and processes that may affect outcomes.

## 1. Introduction

Pediatric chronic pain is a multifactorial experience that impacts psychological, behavioral, and physical functioning and occurs within a larger ecological system [1]. Peers [2], parents, and families can cultivate adaptive outcomes or foster progressive dysfunction [3]. Youth with pain may have fewer friends and avoid social contexts [4], and those who perceive teachers to be unsupportive [5] may avoid school and experience more academic challenges. Lower social functioning mediates the link between physical symptoms and school, and can result in reduced school attendance [2]. Family functioning is so vital that it impacts disability more than pain intensity [3]. Accordingly, chronic pain treatment may warrant attention to multiple systems—and perhaps, particularly so, when youth are feeling isolated within the very systems meant to provide them with support.

Evidence-based pediatric pain interventions include individual and group cognitive behavioral (CBT) and acceptance and commitment therapies (ACT), peer support [6], and parent/family [7] treatment. Therapies target processes such as pain catastrophizing, acceptance, and protective parenting, as well as outcomes such as functional disability, quality of life, anxiety, and depression. Published protocols have been predominantly CBT- or ACT-based, occurring within one individual, family, or group setting. Previous studies have included 6 group CBT sessions [8], 8 individual ACT sessions [9], and 14 youth and 4 parent group ACT sessions [10]. As youth and parent sessions are often held separately, adolescents and families may have little opportunity to meet or be mentored by others with personal experience effectively managing pain [6]. Applying social learning theory to a multifamily setting might foster synergistic changes in the numerous systems impacted by chronic pain and dysfunction [11] and pave a path for a meaningful new wave of treatment.

Evidence based core components of CBT regularly employed to reduce pain, disability, and symptoms include psychoeducation, cognitive reframing, and behavioral activation [12]. Cognitively, pain catastrophizing is often targeted, as focus on unhelpful pain thoughts by youth or caregivers can increase discomfort and dysfunction [13]. Behavioral interventions can also have a significant impact on pain experience and resultant functioning, as activity pacing boosts functioning, exposure mitigates anxiety, pleasant events assuage depression, and decreases in protective parenting attenuate dysfunctional pain behavior. A 6-session CBT group facilitated reductions in pediatric abdominal pain, improved parent confidence to empower their children to use pain coping skills, and increased adaptive behavioral responses among parents themselves [8]. Quality of life and resilience constructs such as acceptance, flexibility, and positive peer/family dynamics, however, were not examined [14]. Investigators consequently invited comparing CBT with other modalities, as well as revising protocols to incorporate acceptance interventions through treatments such as ACT.

Some investigators and clinicians view ACT as a “third wave” of cognitive and behavioral therapies ripe for application to chronic pain conditions. In ACT, physical and emotional distress is viewed as universal and inevitable—to be acknowledged and managed, not evaded or denied [15]. CBT often targets altering how one conceptualizes and copes with distressing conditions in order to reduce symptoms of pain, anxiety, depression, anger, etc. Predominantly focusing on symptom reduction or elimination, however, can perpetuate disability and reduce quality of life. ACT complements and extends beyond CBT by encouraging acceptance of whatever symptoms of discomfort may remain as a means to reduce suffering, and urges the pursuit of meaningful activities even when living with discomfort through concepts such as pain willingness and activity engagement [16]. Interestingly, the more one participates in valued experiences, the more one engages in therapeutic processes known to CBT such as exposure and behavioral activation, subsequently allowing for possible reductions in anxiety, depression, and pain and improvements in quality of life. More research is needed on pain acceptance in diverse functional contexts such as home, school, and the community, and how it may be impacted by adolescent age, family dynamics, and social functioning [16]. 

Pediatric individual and group ACT for pain both have strong empiric backing [17]. ACT components such as acceptance, cognitive defusion, and pursuit of values-based living help youth with pain to increase functional ability, enhance life quality, and lower pain interference [9]. Parents with greater acceptance evidence less catastrophizing and protective parenting [18] that in turn help to foster better outcomes for children. Kanstrup et al. [10] uncovered comparable improvements in pediatric and parent psychological flexibility, pain reactivity, and depression in both individual and group-delivered ACT. Though evidence for family pain interventions is limited [7], therapies with a group context might enable more positive interactions in the home. Treatment research for pediatric chronic pain in families should include development of feasible and acceptable interventions, collection of feedback from multiple responders (e.g., parent, youth, peers), and analysis of both risk and resilience concepts paramount to pediatric health outcomes [19].

Employing multi-family therapy (MFT) in particular might complement pain psychology treatments in ways providers and individual groups cannot, as families are encouraged to acquire skills while also being therapeutic for one another. Asen and Scholz [20] delineate how MFT may benefit youth with behavioral health conditions and their families, including providing the opportunity to empathize, overcome social isolation, promote hope, inspire perspectives, co-discover skills, and reinforce changes in others while gaining confidence to do the same. MFT can enhance real-life interactions within ecological systems (family, friends, school) pertinent to living with pain. The onus is on clinicians and researchers to design interventions that build skills by connecting systemic influences to foster positive changes.

Despite possibilities inherent to group support, very few pain interventions to-date have utilized a multifamily setting. Logan and Simons [11] employed a CBT protocol with a partial MFT format. The program was satisfying, and participants endorsed improved pain, school attendance, mood, and self-esteem; however, peer support dynamics were not examined. Similarly, youth and parent groups met briefly at the end of sessions to review materials, but Noel et al. [8] did not address impact of families meeting as a group. In another realm, Carpenter, Price, Cohen, Shoe, and Pendley [21] designed an MFT to optimize Type 1 Diabetes management via problem-solving. The program was launched to improve treatment access while also providing families with a possibly less stigmatizing alternative to traditional therapy. MFT was acceptable with good preliminary results, yet outcomes of interest for this study—including the effects of in-session support on families—were not assessed, along with other mediators of change such as communication, acceptance, and social connectedness.

Designing a group that includes CBT, ACT, and MFT could improve access to bio-behavioral treatment for pain while also addressing the feeling of isolation in children and their families in various ecological systems (schools, social groups). There is no protocol to-date, however, that emphasizes treatment for symptom management, living despite the symptoms, and fostering system connections to facilitate changes in both pain and function. Therefore, the aims of this investigation were to: (1) describe the engagement in, perceived utility of, and satisfaction with the CAPTIVATE program; and (2) capture progress in key processes relevant to the treatment of chronic pain such as catastrophizing, acceptance, protective parenting, and family dynamics, as well as the outcome variables they are known to impact. Investigators hypothesized that CAPTIVATE would: (1) be well received by families given its goal to facilitate connectedness, cultivate coping, and reinforce vital living despite pain; and (2) enhance psychological, behavioral, and family processes, and associated functional outcomes.

## 2. Materials and Methods

### 2.1. Participants and Procedures

The Stanford University Research Compliance Office for Human Subjects Research and Institutional Review Board (IRB) approved this investigation (protocol ID 29634) beginning 31 January 2014. The project was designed for youth 13–17 years old and their caregivers recently evaluated by or in treatment at the outpatient Pediatric Pain Management Clinic. Physicians, nurse practitioners, and psychologists invited families to participate, and flyers were posted. Those with conditions unsuitable for group (e.g., psychosis; acute depression with safety concerns) and inability to read ≥5th grade English were excluded. Caregivers provided initial medical and demographic data. Youth and parents completed pre- and post-treatment measures on process and outcome variables. Lastly, families provided quantitative and qualitative feedback on program components and dynamics.

CAPTIVATE was conducted as a 9-week program that included weekly concurrent 60 min youth and parent groups led by a licensed pain psychologist or postdoctoral fellow. Each week concluded with an additional 30 min multi-family group led by both providers. While other pain workshops have been designed to be conducted on weekends to increase treatment access (e.g., The Comfort Ability program [22]), resource limitations at our institution prevented this model. As an alternative, the CAPTIVATE program was carried out on weeknights from 5 p.m. to 6:30 p.m. to reduce impact of appointments on school for youth and work responsibilities outside the home for caregivers.

Pain psychoeducation was provided as the foundation to highlight the utility of behavioral health treatments in integrative pain management. CBT modules were presented first to familiarize treatment-naïve participants with emotional, cognitive, and behavioral coping skills while reinforcing these techniques for those with previous pain psychology experience. Participants specifically learned about the impact of feelings on the pain experience, and were empowered to employ biobehavioral strategies (e.g., diaphragmatic breathing; visual imagery; relaxation) to reduce anxiety, sadness, discomfort and distress (e.g., stress, irritability). Participants then reviewed the link between constructive versus less constructive thinking patterns (e.g., pain catastrophizing) when coping with pain, and were encouraged to utilize more helpful thoughts to improve comfort. The value of behavioral activation and engaging in pleasant individual, social, school, and family activities was also encouraged. Midway through the program participants met individually with providers to discuss reflections on the group experience.

The order of intervention delivery (CBT before ACT) was designed to instill basic skills to allow families to feel more comfortable making a paradigm shift from symptom amelioration to living life despite, and even with, discomfort. ACT tenets were then introduced as a model of pain management in which resilience, quality of life, and engagement in valued life activities were emphasized over pain control. Specific concepts such as acceptance for and tolerance of physical and emotional discomfort were discussed, along with the utility of separating self from thoughts (i.e., cognitive defusion). Willingness to engage in activities despite the pain was also emphasized, particularly in the service of pursuing values important to them as youth, caregivers, families, siblings, and friends.

Last, the entire CAPTIVATE protocol included weekly MFT to foster reflections on typical issues faced by families managing chronic pain, such as how to talk about pain in schools, with friends, and with neighbors. The groups also included routine brainstorming to delineate family-based solutions to frequent challenges, such as how to negotiate chore completion and school attendance when pain is elevated. The MFT portion also capitalized on the use of social support to enforce accountability and encourage change. Specific MFT techniques such as cross-fertilizing (i.e., pairing youth with other caregivers) were employed to enhance adolescent understanding of their parent(s) through the lens of other caregivers, and caregiver understanding of their children via the views of other adolescents. Additional module details and techniques are outlined in Table 1.

### 2.2. Engagement, Utility, and Satisfaction (Aim 1)

Quantitatively, engagement was operationalized via retention rate and average session attendance. Utility was assessed on a 0–10 scale regarding perceived improvements in overall functioning (10 = significant progress) and pain (10 = no more pain) at the end of 9 weeks. Satisfaction was rated 0–10, with 10 reflecting 100% satisfaction. Qualitatively, participants were also asked a series of open-ended, audio-transcribed questions the last week of group, including invitations to denote the most helpful parts of the group(s), as well as areas to improve. Families also shared written feedback with similar prompts regarding the impact of CAPTIVATE on socialization, school, personal wellbeing, and family relationship domains. Youth and parent semi-structured interviews and written feedback were then transcribed verbatim and analyzed with QSR International (Americas) Inc. NVivo 11 Pro software (Burlington, MA, USA) for qualitative thematic content. Totals of 14 youth and 20 primary caregivers provided qualitative feedback for analysis. Two research coordinators independently coded each transcript with open coding, allowing for the emergence of themes from the raw data to be grouped into thematic categories. Major themes reported by at least three participants per cohort were delineated. Team discussion with the PI resolved any discrepancies to gain consensus.

### 2.3. Process and Functional Outcomes (Aim 2)

*Pain Catastrophizing*. The pediatric Pain Catastrophizing Scale (PCS) is a valid and reliable tool that assesses catastrophic pain thinking patterns, including rumination, magnification, and helplessness (e.g., “When I have pain, I keep thinking about how much I want the pain to stop”; “When I have pain, it’s terrible and I think it’s never going to get better”) [24]. The parent version assesses thoughts about their child’s pain (e.g., “When my child is in pain, I keep thinking about how much he/she is suffering”; “When my child is in pain, I wonder whether something serious may happen”) [25]. Both include 13 items 0 (Not at all true)–4 (Very true), with three clinical reference categories for youth: low (0–14), moderate (15–25), and high (≥26) [13].

*Pain Acceptance*. The 20-item Chronic Pain Acceptance Questionnaire measures youth [16] and parent proxy [18] acceptance for discomfort (e.g., activity engagement; pain willingness), with sound reliability and validity. Youth CPAQ items are scored 0 (Never True)–4 (Always True) (e.g., “I do things that are important and things that are fun, even though I have chronic pain”) with a similar parent response scale of 0–6 (e.g., “My child is getting on with life no matter what the pain level is”). Higher scores reflect greater chronic pediatric pain acceptance.

*Protective Parenting*. The 26-item Adult Responses to Children’s Symptoms (ARCS) measures what a caregiver does when the child experiences pain. Parents report frequency of behaviors on a scale from 0 (Never) to 4 (Always). The reliable and valid 13-item Protect Scale captured parenting processes such as allowing child to stay home from school due to pain, get out of chores, gain more attention, etc. [26].

*Family Functioning*. The 60-item McMaster Family Assessment Device (FAD) has 12 General Functioning items [27] and measures adaptive (e.g., “We can express feelings to each other”; “Individuals are accepted for who they are”) and maladaptive functioning (e.g., “Making decisions is a problem for our family”). Scores ≥2 on the reliable and valid FAD-GF reflect greater concern regarding family dynamics [3].

*Functional Disability*. The 15-item Functional Disability Inventory (FDI) [28] evaluates difficulty with completing daily activities (e.g., walking, completing chores, staying at school all day) on a scale of 0 (No Trouble)–4 (Impossible). Higher scores on the reliable and valid measure reflect more pain-related disability with 3 clinical reference points: No/Minimal Disability (0–12), Moderate Disability (13–29), and Severe Disability (≥30) [29].

*Quality of Life*. The Pediatric Quality of Life (PedsQL) 4.0 SF15 Generic Core Scales capture School (3 items), Social (3 items), Emotional (4 items), and Physical (5 items) functioning over the last month in youth 13–18 years on a 0 (Never) to 4 (Almost Always) Likert scale. Raw scores are transformed to a scale of 0–100, with 100 indicative of exceptional quality of life in that domain. School items query problems paying attention in class, forgetting things, and keeping up with schoolwork. Social items query challenges related to getting along with other teens, teens wanting to be friends with the respondent, and being teased. The Short-Form has adequate reliability [30].

*Health and Psychological Functioning*. The Patient Reported Outcomes Measurement Information System (PROMIS) evaluates outcomes for youth 8–17 years and parent proxy reports (see www.nihpromis.org) over the last week. The Short Forms (V1.0) were employed. Responses of 0 (Never/Not Able to Do) to 4 (Almost Always/No Trouble) are transposed to a T-score (*M* = 50; SD = 10). Higher T-scores reflect more symptoms, with clinical categories indicative of elevated (≥60) to significantly elevated (≥70) symptoms. Validity and change sensitivity has been established [31] for current study domains: (1) Pain Interference (8 items): impact on physical, psychological, social functioning; (2) Fatigue (10): impact on activities; (3) Anxiety (8): fears, nervousness; and (4) Depressive Symptoms (8 youth; 6 parent): negative mood, perception, and cognition.

*Current Pain Intensity*. Youth rated pain on a 0 (None)–10 (Worst pain) numeric rating scale [32].

*Data Analysis Plan for Outcomes*. Two participants were dropped from analyses due to significant missing data (one did not complete baseline measures and the other did not turn in final measures). This left 15 youth and 15 primary caregivers with pre- and post-intervention data for all questionnaires except the PedsQL, where one cohort did not answer post-treatment, leaving 8 youth and 8 primary caregivers for analysis. All PCS, CPAQ, ARCS, and FDI questionnaires were answered in full except in the following cases, where scores were computed using available data: PCS (1 parent answered 11/13 items), CPAQ (1 child and 8 parents answered at least 18/20), ARCS Protect (2 parents answered at least 13/15), FDI (3 children and 4 parents answered 14/15). PROMIS surveys with missing data were scored and normalized by number of questions answered and converted to T-scores, per standard procedures. A bootstrap method analogous to a 2-tailed paired *t*-test was used to assess pre- to post-intervention changes, as some datasets were too small to reasonably verify normality [33]. The mean of all pre-post differences was defined as the test statistic for each survey. These differences were multiplied by −1 or 1 to randomize direction of change, the mean was recomputed, and the process repeated 100,000 times to form a distribution of the test statistic under the null hypothesis that the effect of treatment on scores was no better than expected by chance. *p*-values were defined as the fraction of randomized values that were at least as extreme as the actual statistic or its reflection across the mean of the null distribution, then adjusted for number of surveys using the Benjamini-Hochberg BH step-up procedure [34]. The mean of pre-post differences divided by the standard deviation provided Cohen’s *d* effect sizes.

## 3. Results

### 3.1. Program Engagement, Utility, and Satisfaction (Aim 1)

CAPTIVATE consented 18 families for participation, and ran for three cycles with 5, 7, and 6 families in each cohort. A pain psychologist recruited twelve families (67%) after they reported a goal to connect with and learn from other youth and families living with chronic pain. A physician recruited five families treatment-naïve to pain psychology after an initial pain clinic evaluation, and one family (6%) was self-referred via flyers posted. Overall retention was 94% after one family perceived the child was too disabled to participate and dropped out after week 1.

The remaining 17 youth (*M*_age_ = 15.4; 60% female; 80% Caucasian) had pain for an average of 22 months (range 6–60). Conditions included Headache (40%), Musculoskeletal (33%), Abdominal (20%), and Other (7%) Pain. Academically, they were enrolled in Public (47%), Combination (independent study/home-hospital), or Private (13%) School. More than half of participants who enrolled (65%) had previous pain psychology treatment. Caregivers (*M*_age_ = 49.3; 80% mothers) were college-educated (80%) with private health insurance (88%).

Consistent with Aim 1 and hypotheses, youth and their parents were able to engage throughout the program. Youth attended an average of 7.8 sessions and parents an average of 7.6 sessions. Participants also endorsed moderate perceived utility for the program in terms of helping to improve overall pain (*M*_youth_ = 4.2; *M*_parent_ = 4.1) and functioning (*M*_youth_ = 4.7; *M*_parent_ = 5.8), and high levels of CAPTIVATE satisfaction (*M*_youth_ = 7.6; *M*_parent_ = 8.2). Qualitatively, they also reported several themes specific to their participation in this multi-family group intervention:

Theme 1: Social Support, Empathy, and Views. A majority of youth (57%) and caregivers (80%) cited their groups as validating and supportive, with youth (21%) and parents (30%) noting benefit from hearing cohort viewpoints. One parent stated: “Just hearing that we weren’t alone from the very first day...meant a lot. It changed the whole family dynamic at home.” One youth shared: “I really benefited from the relationships that I formed. It’s incredibly helpful to have people who understand what I’m going through and have very similar experiences.”

Theme 2: Pain Management Coping via CBT and ACT. Learning skills and adaptive psychological processes was helpful for 50% of youth, who emphasized utility of cognitive coping (29%) and pain acceptance (21%). One youth stated, “I’ve been using cognitive coping and positive behavior change actively since we went over them, and they’ve been very helpful in my journey.” Another highlighted a shift in acceptance to pursue values: “Yes, I will experience pain, but I will do it anyway…my mind frame has shifted.” Parents cited psychoeducation (35%), acceptance and values (35%), and reducing daily pain queries (15%) as constructive, with one sharing, “It was a big change to go from focusing on pain to focusing on functioning.”

Theme 3: Utility of Multi-Family Therapy (MFT) Group. Youth (64%) reported that MFT improved family communication and understanding. Social modeling, encouragement (21%), and exposure to other parent perspectives (21%) were also useful. Increased understanding of their child by hearing from others was noted by parents (35%), along with validation for their family pain management experience (70%) in part due to hearing other families’ stories (20%). 

Theme 4: General Feedback and Areas to Improve. Youth identified improvements in overall socialization (50%) and school functioning (43%), while 65% of parents denoted increased personal wellbeing. To enhance CAPTIVATE, 45% of youth and parents reported wanting more and/or longer sessions, and parents added a desire for refresher courses. See Table 2 for additional details.

### 3.2. Process and Functional Outcomes (Aim 2)

Consistent with the second aim and hypotheses, analyses revealed significant changes with large effect sizes in youth pain psychological processes such as decreased catastrophizing (Cohen’s *d* = 0.8) and increased acceptance (*d* = 1.0) (BH-*p* < 0.05). Regarding functional and symptom outcomes, youth endorsed significant reductions in functional disability (*d* = 0.8), increased social (*d* = 1.0) but not school (*d* = 0.5) quality of life, and less pain interference (*d* = 1.1), fatigue (*d* = 0.7), and anxiety (*d* = 0.6) (Table 3).

In terms of parent psychological and behavioral processes, caregivers similarly endorsed statistically significant (BH-*p ≤* 0.05) changes in chronic pain acceptance on behalf of their child with large effect size (*d* = 0.9) as well as reduced protective behaviors (*d* = 0.9). Changes in parental catastrophizing were of medium effect (*d* = 0.5) but not significant (BH-*p* = 0.11). Regarding outcomes, parents also reported decreased functional disability (*d* = 0.9). Unlike their children, parents did not endorse statistically significant changes in pediatric pain interference (BH-*p* = 0.054; *d* = 0.6), fatigue (BH-*p* = 0.054; *d* = 0.6), or anxiety (BH-*p* = 0.11; *d* = 0.5), but did note decreases in proxy depressive symptoms (*d* = 0.9; BH-*p <* 0.05). Last, parents endorsed family dynamic changes on the Family Assessment Device with small effect (*d* = 0.2) that were statistically insignificant yet in the expected direction, as the mean score shifted to just under the clinical cut-point of 2.0 (Table 4).

Post-hoc analyses regarding demographic (age, sex), medical (pain intensity, duration, previous pain psychology), and intervention (number of sessions attended) effects on outcomes revealed no significant relationships with age, pain duration, or session attendance. There were some effects for gender on pain catastrophizing and anxiety, as females showed more improvement. Compared to those with previous pain psychology treatment, those with none (35%) reported greater improvements in psychosocial and school quality of life.

**Figure 1:**
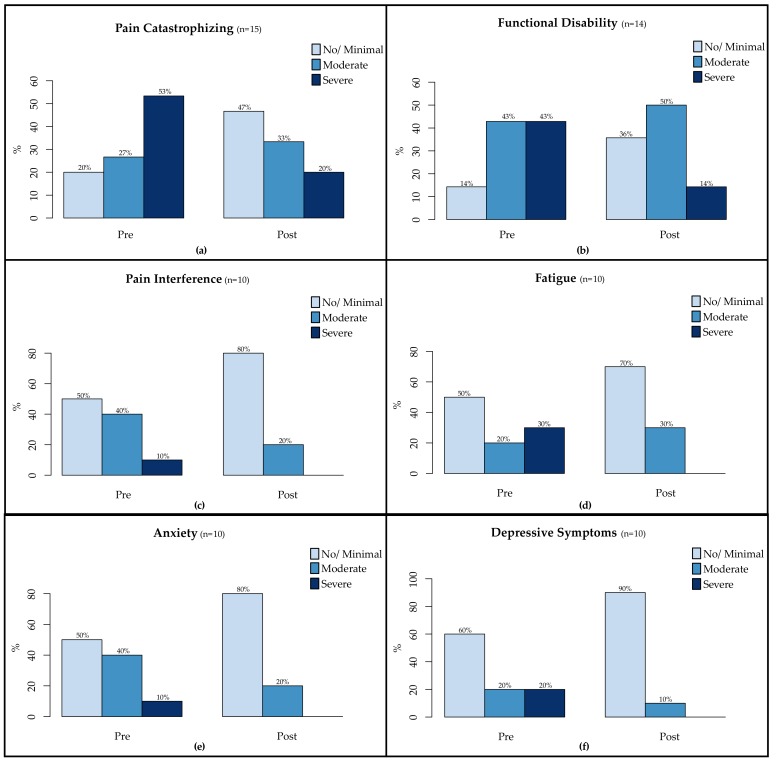
Adolescent Self-Report of Pre-to Post-CAPTIVATE Changes: (**a**) Pain Catastrophizing; (**b**) Functional Disability; (**c**) Pain Interference; (**d**) Fatigue; (**e**) Anxiety; and (**f**) Depressive Symptoms.

Some statistically significant results also translated into clinically meaningful shifts from pre- to post-intervention (Figure 1). In terms of psychological processes, the percentage of youth who were low in pain catastrophizing (raw score ≤ 14) increased from 20 to 47%. Regarding functional outcomes, the percentage of youth who endorsed no/minimal functional disability (raw score ≤ 12) increased from 14 to 36%. Symptom relief was also captured on all PROMIS measures, as the percentage of youth who endorsed no/low symptoms (T-score < 60) increased from 50 to 80% for pain interference, 50 to 70% for fatigue, 50 to 80% for anxiety, and 60 to 90% for depressive symptoms.

## 4. Discussion

Dynamic pediatric chronic pain management involves treatment(s) of youth cognitions, behaviors, emotions, and physical symptoms at individual and group-levels along with their family systems [35]. Optimal interventions concurrently address psychological processes (e.g., acceptance) that promote adaptive functioning, quality of life, and resilience [14] within larger ecological systems [1]. The current program encouraged adaptive skills similar to many CBT and ACT protocols, yet built on traditional treatments with systematic provision of peer, parent, and family group support for those feeling isolated in the experience of chronic pain. Adoption of a multi-family component in particular might heighten outcome improvements through social learning, helping individuals to manage authentic interactions within their families, schools, and communities thereafter with more confidence [20].

Consistent with the first aim and hypotheses for this investigation, quantitative findings revealed high CAPTIVATE program engagement, perceived utility, and satisfaction, which compose markers of intervention acceptability [36]. Qualitative feedback also captured participant appreciation for group-derived validation, empathy, and facilitated communications. High retention, attendance, and positive treatment experiences may have related in part to alliance with the providers, who emphasized patient- and family-centered communication, collaboration, goal setting, and pain education [37] in the service of cultivating sound health and pain-specific outcomes [38,39]. The preliminary results from this program must therefore be replicated with a case-control design led by other clinicians with larger samples in order to determine the value-added contribution of the specific content and processes inherent to the CAPTIVATE design.

Consistent with the second aim and hypotheses, participants endorsed significant improvements in processes (e.g., pediatric and parent acceptance; pediatric catastrophizing; protective parenting) and functional outcomes (e.g., disability; pain interference) per large effect sizes and reductions in multiple clinically relevant domains. Larger effects may have occurred for these domains over, for example, anxiety symptoms (which nonetheless evidenced medium effect) because CBT and ACT modules targeted pain catastrophizing and acceptance over treatment of more diffuse anxiety and depressive symptoms. Additional investigation is warranted to tease apart intervention components, and how effects are measured over time. Treatment principles such as behavioral activation and living life despite discomfort also appeared to foster changes in school and social systems as key outcomes in chronic pain [1,11]. The presence of modest (*d* = 0.6), non-significant improvements in school quality of life coupled with qualitative perception that functioning did improve similarly compels need for future research involving school-specific collaborations and objective data collection. The presence of significant and meaningful social changes highlights the possible importance of developing a group to foster interpersonal confidence for youth with chronic pain when with other peers and friends.

The occurrence of functional changes in the absence of any significant changes in pain level in an intervention with a family component was also congruent with previous literature highlighting the impact of family dynamics on functional changes over and above pain amelioration [3]. In this regard, findings also build on and address some limitations of previous pediatric studies involving multiple families [11,21]. Law et al. [7] completed a review of parent- and family pain treatments, including those targeting interactions within social, work, and medical contexts. As with this study they found small parenting enhancements, but interventions did not lead to quantitatively captured improvements in family functioning. Notably, their review did not include qualitative outcomes. Results from this investigation captured qualitative data with markers of effective MFT [20], including appreciation for in-session dynamics such as: validation, increased perspective, and exposure to social modeling. Preliminary enhancements in family communication, social connectedness, and other ecological systems (e.g., school) were also reported, and while not statistically significant on the FAD, family dynamic process improvements were in the expected direction.

Independent of measurable findings, a meaningful take-home point remains that pediatric pain as a social and family experience is more layered than a number on a scale or measure, and may require support not available solely through individual therapy sessions. Empathic peer, parent, and family-to-family encouragement may provide a surrogate ecological system through which individuals gain confidence and learn to improve authentic relationships. Qualitatively, youth endorsed improved social life and noted better school functioning, consistent with the idea that peer support may protect youth from the detrimental effects of pain on school [2]. Similarly, caregivers indicated better wellbeing despite lack of targeted mental health support. Extending beyond traditional services MFT may have helped to cultivate a group synergy that buoyed improvements in process and outcomes. The MFT context may have also facilitated changes within the home, as youth identified improved communication, and parents reported better understanding of their children because of hearing stories from other youth and families.

The importance of offering the program after school and standard work hours (5 p.m.) and adopting flexible inclusion criteria (e.g., various pain conditions; exposure to previous treatment) reflect other notable take-home factors. This heightened general access to specialty pain psychology services for busy families while also providing a treatment that none had had within a group environment. The 9-week program was time-intensive, however, and pain clinics might also consider offering comprehensive review of pain coping strategies in an accessible format that is time-limited, such as The Comfort Ability one day workshop [22]. Although flexible enrollment may improve general access to care, screening for baseline pain condition, developmental status, and less constructive behaviors (e.g., counterproductive coping such as avoidance or substance use; embellishing issues for attention) is also recommended. Promoting acceptance of pain might not bode well, for instance, for those with hope for remission (e.g., youth with post-concussive headaches).

Some families may also be better suited for CAPTIVATE than others. Those who prefer an individually tailored pain psychology approach are not likely to enroll, but those “on the fence” who do may not gain as much from the group, especially if they evidence certain characteristics. For example, youth and parents more comfortable within and motivated by prosocial environments might benefit more from MFT than those managing vulnerabilities (e.g., impairing social anxiety) or family stressors (e.g., divorce, reduced resources) that may limit engagement. Participants who have had negative clinic interactions, deny emotions as integral to the pain experience, or engage in unconstructive or competitive talk about pain (i.e., who is most disabled and how) [40] may also be iatrogenic to the group dynamic. As with most group therapies, communication patterns among participants were monitored week to week. Less constructive discussions in parent (e.g., dissatisfaction with providers; insurance policies) or teen groups (e.g., complaints about treatments that were less helpful) were rare, but reframed and redirected as indicated.

The practicalities of a busy clinical practice contributed to limitations of this investigation. Pain physicians and nurse practitioners helped with participant recruitment; however, they were not asked to systematically track who was approached, who accepted/declined, and why. Therefore, a participant flow diagram as one component of feasibility was not available. A conservative methodology that was employed to prevent spurious findings within a small sample and multiple analyses still uncovered promising preliminary results. Nevertheless, the results have limited generalizability and must be interpreted with caution due to: non-random participation; issues with missing data and lack of follow-up data; small sample size; and the lack of a control/comparison group. Applicability to the general population is further limited by the fact that participants who enrolled in the program predominantly came from households with college education and private insurance. Causality cannot be inferred at this time as changes could be due to time or other interventions. Should a randomized control trial be launched, data would be methodically traced to inform a CONSORT diagram. Larger samples would also enable investigation of moderating (e.g., sex effects on catastrophizing) and mediating (e.g., acceptance) variables to augment understanding of therapeutic mechanisms of transformation in pain groups [41].

Multiple future directions might be considered. The differential yield of CBT, ACT, and MFT has not yet been studied in a pediatric sample. A component analysis specific to treatment principles (e.g., behavioral activation; cognitive defusion) and exploring the possible benefit of combining therapies is warranted, as is consideration of the financial implications of individual/family versus MFT. Expanding the CAPTIVATE intervention to include support for systems such as schools [11] and siblings [12] might also be constructive. Youth with pain endure more bullying and school absenteeism, but effects are attenuated in the context of supportive teachers [5]. Mentors who provide pain education and model adaptive coping help youth with pain to better manage other environments by fine-tuning coping skills [6]. Offering teachers similar pain education to better prepare them to encourage their students’ pain coping may be helpful. Participants in this study shared that when teachers felt more encouraging they had more confidence attending and being within their school environment despite discomfort. Due to home environment and genetics, siblings of youth with pain are also at risk of developing pain, behavioral health vulnerabilities, and challenges in their school and social systems [42]. Multi-family work may allow closer focus on the interconnected nature of family relationships—including to pain—and help to delineate factors that foster adjustment for all affected individuals.

## 5. Conclusions

Provision of sound behavioral health support for youth with chronic pain and their families includes attunement to their lives within a larger ecological system. Utilizing social contexts that encourage high-quality peer relationships and support may help to foster adaptive outcomes among adolescents and their parents, and further understanding of family-level risk and resilience factors [43]. Results from this investigation suggest that integrating evidence-based modalities such as CBT and ACT and couching them within peer, parent, and multi-family settings can be an engaging, acceptable, and potentially useful treatment.

## Figures and Tables

**Table 1:** Intervention Session Content and Process.

Week	Session Title	Description
Week 1	Introduction to pain management	Reviewed goals and expectations of the program, and provided pain psychoeducation. Highlighted the biopsychosocial model and gate control theory of pain [23] as frameworks for pain management.
Week 2	Introduction to CBT: Coping with feelings promotes comfort	Introduced coping tools and strategies for managing feelings and discomfort. Groups discussed how emotions impact pain and brainstormed ways to address distressing emotions. Strategies were practiced in session.
Week 3	Cognitively-based coping	Emphasized the importance of monitoring and altering thoughts to increase well-being. Discussed maladaptive thought patterns such as pain catastrophizing. Introduced more adaptive ways of thinking about management of discomfort.
Week 4	Creating a CAPTIVATING life through positive behavior change	Discussed skills learned for behavioral change. Practiced goal-setting. Presented adaptive and maladaptive behaviors for pain management in caregiver group. Youth and caregivers jointly participated in solution-oriented thinking regarding the topic of school attendance, and siblings welcomed.
Week 5	Individual check-in	Each participant met with a clinical psychologist for 50 min.
Week 6	Introduction to ACT: Core principles and cognitive defusion	Introduced ACT as an intervention that aims to increase psychological acceptance and flexibility, and its six core principles, including cognitive defusion. Encouraged youth and caregivers to employ the principles and identify values.
Week 7	Acceptance and willingness	Introduced concepts of acceptance and willingness as an alternative to experiential avoidance. Reviewed mindfulness techniques emphasizing attention on an immediate experience and feelings of openness and acceptance.
Week 8	Capturing life and acting according to values	Identified values and committing to them through action. Discussed solely seeking to control and/or avoid pain versus choosing to pursue a valued, meaningful life.
Week 9	Review and graduation	Discussed teen, parent, and family aspects of the group experience. Members provided preliminary feedback. Celebrated participation, connections, and enhanced CBT and ACT skill use among families.
Weeks 1–8	Multi-family concepts and techniques [20]	During weekly family sessions employed the 5-step model of reflecting problematic interactions and communications, assessing opinions, inviting feedback, evaluating change goals, and encouraging experimentation and action. Specific techniques included: connecting, stimulating and woodpecking (selecting and discussing specific problematic family interactions), intensifying (between families), cross-fertilizing (connecting youth with different parents), circling (orbiting around families), and retreating (leaving families to themselves).

**Table 2:** CAPTIVATE Qualitative Themes.

	Youth (14)	Parent (20)
Most Helpful Parts: Teen Group		
Validation, empathy and social support	57%	
Coping skills	50%	
Cognitive techniques	29%	
Peer perspectives and insights	21%	
Pain acceptance	21%	
Most Helpful Parts: Parent Group		
Validation, empathy and social support		80%
Increased communication and family relationships		75%
Better understanding of child (through hearing other parents’ experiences)		20%
Pain psychoeducation		35%
Shared perspectives and experiences		30%
Stop or minimize daily pain queries		15%
Most Helpful Parts: Family Group (per Youth)		
Improved communication and understanding	64%	
Normalization	29%	
Hearing other parents’ perspectives	21%	
Exposure to social modeling/encouragement	21%	
Most Helpful Parts: Family Group (per Parents)		
Validation and social support at the family level		70%
Hearing from others increased understanding of child		35%
Emphasis on pain acceptance and family values		35%
Safe place for sharing stories		20%
General Program Feedback		
Helped with socialization	50%	
Helped with school functioning	43%	
Helped with personal wellbeing		65%
Helped with job situation		20%
Areas to Improve		
Longer and/or more sessions	45%	45%
Refresher courses/follow-up sessions		25%
Promote materials review and homework completion		15%

**Table 3:** Pre and Post-CAPTIVATE: Youth Outcomes.

Outcome	Pre-Mean (SD)	Post-Mean (SD)	Effect Size (Cohen’s *d*)	ES 95% CI	*p*-Value (BH)
Pain Catastrophizing *	24.97 (9.04)	16.83 (10.01)	0.78	0.37, 1.40	0.024
Pain Acceptance *	38.63 (11.67)	49.37 (14.16)	−0.97	−1.60, −0.70	0.003
Functional Disability *	25.67 (10.02)	17.50 (11.56)	0.80	0.45, 1.40	0.012
Social Functioning *	83.33 (16.67)	93.75 (11.56)	−0.98	−1.90, 0.54	0.050
School Functioning	27.08 (22.60)	48.44 (27.63)	−0.55	−1.20, 0.05	0.175
Psychosocial Health	48.91 (17.55)	64.54 (13.63)	−0.75	−1.5, −0.31	0.096
Pain Interference *	60.98 (5.79)	53.37 (7.73)	1.16	0.77, 1.90	0.003
Fatigue *	62.73 (9.32)	53.83 (10.64)	0.72	0.19, 1.80	0.037
Anxiety *	56.89 (11.63)	50.73 (11.00)	0.64	0.16, 1.40	0.048
Depression	56.78 (9.87)	52.69 (8.76)	0.37	−0.13, 1.10	0.175
Current Pain Intensity	4.86 (2.69)	4.04 (2.62)	0.65	0.22, 1.20	0.054

* BH-adjusted *p* ≤ 0.05. ES—Effect size. CI—Confidence interval.

**Table 4:** Pre and Post-CAPTIVATE: Parent Outcomes.

Outcome	Pre-Mean (SD)	Post-Mean (SD)	ES (Cohen’s *d*)	ES 95% CI	*p*-Value (BH)
Pain Catastrophizing	23.50 (9.32)	19.30 (11.47)	0.51	0.00, 1.50	0.118
Pain Acceptance *	47.00 (14.05)	61.23 (16.32)	−0.90	−1.90, −0.40	0.019
Functional Disability *	25.83 (9.36)	18.90 (8.14)	0.92	0.46, 1.70	0.019
Social Functioning	79.60 (16.20)	81.50 (13.68)	−0.09	−0.96, 0.64	0.787
School Functioning	36.50 (11.70)	44.80 (15.39)	−0.48	−1.10, 0.28	0.203
Psychosocial Health	50.30 (14.20)	59.40 (6.96)	−0.57	−1.60, 0.06	0.139
Pain Interference	64.13 (4.98)	58.33 (9.23)	0.63	0.24, 1.10	0.054
Fatigue	66.47 (5.94)	60.67 (8.47)	0.62	0.26, 1.10	0.054
Anxiety	64.33 (9.46)	58.67 (8.86)	0.50	0.04, 0.99	0.118
Depression *	63.60 (9.68)	53.50 (8.71)	0.90	0.38, 1.80	0.019
ARCS Protect *	1.60 (0.64)	1.18 (0.55)	0.89	0.44, 1.60	0.019
Family Assessment	2.01 (0.53)	1.94 (0.68)	0.19	−0.34, 0.72	0.549

* BH-adjusted *p* ≤ 0.05. ES—Effect size. CI—Confidence interval. Parent PROMIS measures are proxy reports.
